# NBAS deficiency due to biallelic c.2809C > G variant presenting with recurrent acute liver failure with severe hyperammonemia, acquired microcephaly and progressive brain atrophy

**DOI:** 10.1007/s11011-021-00827-z

**Published:** 2021-08-24

**Authors:** Patryk Lipiński, Milena Greczan, Dorota Piekutowska-Abramczuk, Elżbieta Jurkiewicz, Agnieszka Bakuła, Piotr Socha, Irena Jankowska, Dariusz Rokicki, Anna Tylki-Szymańska

**Affiliations:** 1 Department of Pediatrics, Nutrition and Metabolic Diseases, The Children’s Memorial Health Institute, al. Dzieci Polskich 20, 04-730 Warsaw, Poland; 2 Department of Medical Genetics, The Children’s Memorial Health Institute, Warsaw, Poland; 3 Department of Diagnostic Imaging, The Children’s Memorial Health Institute, Warsaw, Poland; 4 Department of Gastroenterology, Hepatology, Feeding Disorders and Pediatrics, Children’s Memorial Health Institute, Warsaw, Poland

**Keywords:** NBAS deficiency, Recurrent acute liver failure, Acquired microcephaly, Brain atrophy, Reye syndrome

## Abstract

Biallelic pathogenic variants in the neuroblastoma amplified sequence (*NBAS*) gene were firstly (2015) identified as a cause of fever-triggered recurrent acute liver failure (RALF). Since then, some patients with NBAS deficiency presenting with neurologic features, including a motor delay, intellectual disability, muscular hypotonia and a mild brain atrophy, have been reported. Here, we describe a case of pediatric patient diagnosed with NBAS deficiency due to a homozygous c.2809C > G, p.(Pro937Ala) variant presenting with RALF with severe hyperammonemia, acquired microcephaly and progressive brain atrophy. Not reported in the literature findings include severe hyperammonemia during ALF episode, and neurologic features in the form of acquired progressive microcephaly with brain atrophy. The latter raises the hypothesis about a primary neurologic phenotype in NBAS deficiency.

## Introduction

In 2015, biallelic pathogenic variants in the neuroblastoma amplified sequence (*NBAS*) gene were identified as a cause of fever-triggered recurrent acute liver failure (RALF) (Haack et al. 2015). Staufner et al. (2020) have recently reported results of an international, multicenter study involving 110 patients, including novel and previously published patients. This study had shed a new light on the pathomechanism and expression of NBAS deficiency. Not only liver but also other organs and systems, including the skeletal, immunological and central nervous system, could be affected. Regarding neurologic features, a motor delay, intellectual disability, and muscular hypotonia were observed in some patients (Balasubramanian et al. 2017; Capo-Chichi et al. 2015; Haack et al. 2015; Li et al. 2017; Staufner et al. 2016, 2020; Suzuki et al. 2020). MRI of the brain was normal in the majority of patients while a mild brain atrophy in some of them was reported. Hepatic encephalopathy with hyperammonemia were also transiently observed in some patients secondarily during ALF. However, the natural history of NBAS deficiency, especially regarding the neurologic phenotype, is not known.

Here, we describe a case of a pediatric patient diagnosed with NBAS deficiency due to a homozygous c.2809C > G, p.(Pro937Ala) variant presenting with recurrent acute liver failure with severe hyperammonemia, acquired microcephaly and progressive brain atrophy.

## Case report

The patient was the third child of nonconsanguineous Polish parents born at 39 weeks of gestation by a spontaneous delivery with a birth weight of 3,000 g and head circumference 33 cm (10–25 pc). At 2 months of age head circumference was 37 cm (10 pc). A psychomotor development was normal till 10 months of age. At this age, she underwent the respiratory tract infection with high fever. Recurrent vomiting and increasing apathy within 2 days after the onset of fever were observed and the child was admitted to hospital. At baseline, laboratory results revealed the presence of anemia (Hgb 6.2 g/dl), thrombocytopenia (PLT 60 × 10^3^/uL), elevated serum transaminases (ALT 7725 IU/L, AST 3024 IU/L), hyperbilirubinemia (total serum bilirubin 1.8 mg/dl, direct serum bilirubin not tested), coagulopathy (INR 3.4) and hyperammonemia (serum ammonia 572 umol/l). She was transferred to the pediatric intensive care unit because of hepatic encephalopathy and acute kidney injury.

The greatest degree of elevation of serum transaminases (ALT 7259 IU/L and AST 12,246 IU/L) was noted on the 2^nd^ day of hospitalization. Severe coagulopathy (INR 9.0) and increasing parameters of cholestasis reaching up to total serum bilirubin 6.0 mg/dl with direct serum bilirubin 5.1 mg/dl were noted on the 3^rd^ day of hospitalization. Serum ammonia concentrations were observed elevated (range 572–662 umol/L) during first six days of hospitalization (no ammonia scavengers were administered).

**Fig. 1:**
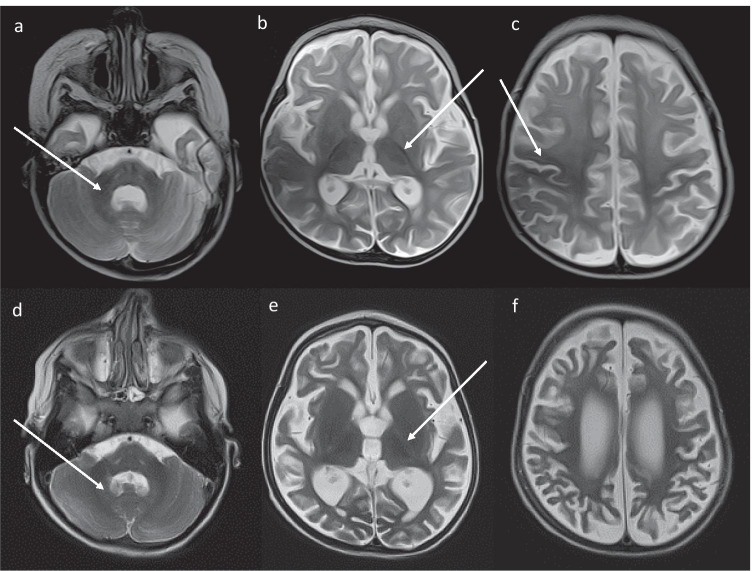
Upper row – MR brain examination at the age of 11 months, axial T2-weighted images. Hypointensity of the pons and cerebellar peduncles (**a**), posterior limbs of internal capsules (**b**), corpus callosum (not shown), perirolandic cortex (**c**) are visible, indicated myelination. Diffuse hyperintensity of the cerebral white matter of both hemispheres is seen. Mild enlargement of the ventricular system, enlargement of Sylvian fissures, subarachnoid spaces along the convexities and anterior interhemispheric falx are demostrated. Bottom row – MR brain examination at the age of 3 years, axial T2-weighted images. Hyperintensive signal of hilum of dentate nuclei (**d**) and posterior limbs of internal capsules (**e**) suggested degeneration of myelin. Progression of the atrophy of the cerebral hemispheres with severe cortical and subcortical atrophy (**e**, **f**)

A fully recover of liver function was noted on the 20^th^ day of hospitalization. Brain MR at that time revealed the presence of extensive symmetrical atrophic cortico-subcortical changes of both hemispheres of the brain (Fig. 1). The patient was discharged home on the 70^th^ day of hospitalization because ALF was complicated by sepsis.

At the age of 3 years, the patient developed the 2^nd^ episode of respiratory tract infection with fever. She was hospitalized within 6 days after the onset of fever due to apathy and weakness in our Institute; no antypiretics were administered ambulatory because of Reye syndrome suspicion. The patient’s head circumference was 44,5 cm (below 3^rd^ pc), height was 94,5 cm (25^th^ pc) and body weight was 12,5 kg (10^th^ pc). Neurological examination revealed the psychomotor retardation and muscle hypotonia. At baseline, laboratory results revealed the presence of elevated serum transaminases (AST 3300 IU/L, AST 1520 IU/L), cholestasis (total serum bilirubin  2.0 mg/dl and direct serum bilirubin 1.0 mg/dl), coagulopathy (INR 1.8). Normal serum ammonia concentration was noted. The patient received parenteral 10% glucose infusion during all hospitalization. The follow-up brain MR examination showed progression of the atrophy of the cerebral hemispheres with severe cortical and subcortical atrophy (Fig. 1). A fully recover of liver function was noted on the 8^th^ day of hospitalization.

All known causes of pediatric ALF, including inborn errors of metabolism, were ruled out. TruSightOne NGS panel containing 5000 genes (including 33 genes associated with acute liver failure development) was commenced. There have been found *NBAS* (RefSeq: NM_015909.3, NP_056993.2) c.2809C > G p.(Pro937Ala) variant, in a homozygous state. The variant was confirmed by Sanger sequencing to be inherited by biparental transmission.

During control visit in the Outpatient Clinic, at 4 years of age, she was able to sit independently, walk with help, speak simple words. Ophthalmologic examination revealed no abnormalities; visual evoked potentials test is planned. Normal serum concentration of immunoglobulins G, A, M was noted. Echocardiography revealed normal structure of the heart.

## Discussion

In the study we reported an additional patient with fever-triggered recurrent ALF associated with NBAS deficiency. Not reported findings include severe hyperammonemia during ALF episode, and neurologic features in the form of acquired microcephaly and progressive brain atrophy.

Staufner et al. (2020) have recently published results of an international, multicenter study defining clinical subgroups and genotype–phenotype correlations in NBAS-associated disease across 110 patients. Three subgroups related to the affected region of the NBAS protein that differed significantly regarding main clinical features were explored, including Sec39 (predominant liver phenotype with recurrent acute liver failure), C-terminal (multisystemic phenotype with the presence of short statute, skeletal dysplasia, immunological abnormalities, Pelger-Huët anomaly and optic nerve atrophy), and β-propeller (combined phenotype with RALF and multisystemic phenotype). The c.2809C > G (p.Pro937Ala) variant identified in our patient, was located in the region coding for the Sec39 domain, thus NBAS deficiency should have a predominant liver phenotype. However, the presence of an acquired microcephaly and progressive brain atrophy questions this hypothesis.

What’s more, there have been reported only 1 patient, found to be compound heterozygous for c.2809C > G, p.(Pro937Ala) and c.([5138 + 1_5139-1]_7116del), p.(?) but no abnormalities of the central nervous system were observed (Staufner et al. 2020).

Motor and cognitive development was normal in the most of reported patients with NBAS deficiency (Balasubramanian et al. 2017; Capo-Chichi et al. 2015; Haack et al. 2015; Li et al. 2017; Staufner et al. 2016, 2020; Suzuki et al. 2020). Neurologic features, including a mild hyperammonemia, encephalopathy and mild MRI abnormalities (non-progressive brain atrophy with normal results of ^1^H‐MRS studies) observed in some reported patients could be explained as a sequelae of ALF, however a primary neurological phenotype cannot be ruled out (Balasubramanian et al. 2017; Capo-Chichi et al. 2015; Haack et al. 2015; Li et al. 2017; Staufner et al. 2016, 2020; Suzuki et al. 2020). The presence of an acquired and progressive microcephaly with brain atrophy in our patient raises the hypothesis about a primary neurologic phenotype in NBAS deficiency. On the other hand, brain atrophy could be secondarily to acute hepatic hyperammonemic encephalopathy (HE), however this phenomenon is reversible in the course of HE while the progression of brain MR suggests a primary central nervous system (CNS) involvement.

Liver involvement in NBAS deficiency is characterized by recurrent episodes of acute liver failure (liver crises) with normalization of liver function between crises Balasubramanian et al. 2017; Capo-Chichi et al. 2015; Haack et al. 2015; Li et al. 2017; Staufner et al. 2016, 2020; Suzuki et al. 2020). Based on our case report, we could speculate that the CNS involvement is independent of the liver dysfunction.

An early and consequent administration of antipyretics together with an anabolic energy management (high glucose and lipids) proved to be highly beneficial on liver function recovery but it seems to have no effect on the course of CNS involvement.

The long-term outcome in NBAS deficiency is unknown. Suzuki et al. have recently reported the oldest patient (34-year-old) showing a progressive course of disease (Suzuki et al. 2020). The patient presented with RALF until an early childhood, epilepsy diagnosed at 12 years of age, progressive intellectual disability and liver fibrosis with portal hypertension at 20 years of age.

Giving the similarities between the clinical presentation of NBAS deficiency and Reye syndrome (defined as an acute non-inflammatory encephalopathy with acute liver failure) we could speculate about the classification of NBAS deficiency as a Reye-like syndrome (Schrör 2007). It is obvious that a primarily genetic background as the cause of Reye syndrome, was not identified or even considered in the 1960s and 1970s when the majority of cases were reported (Schrör 2007). Nowadays, such a genetic background is an explanation for recurrent Reye-like symptoms triggered by infections/fever and should be considered. Thus, every patient presenting with Rey-like symptoms should undergo molecular analysis, especially including *NBAS* gene.

## Data Availability

All data generated or analyzed during this study are included in this article.
